# Emerging applications of artificial intelligence for obstetric ultrasound: A scoping review

**DOI:** 10.1002/ijgo.70789

**Published:** 2026-01-09

**Authors:** Vani Gupta, Nicole Santos, Sara Della Ripa, Dilys Walker

**Affiliations:** ^1^ University of California, Berkeley Berkeley California USA; ^2^ Institute for Global Health Sciences University of California, San Francisco San Francisco California USA; ^3^ Department of Obstetrics, Gynecology and Reproductive Sciences University of California, San Francisco San Francisco California USA

**Keywords:** artificial intelligence, fetal, maternal, obstetric, scoping, ultrasound

## Abstract

**Background:**

The WHO recommends that all pregnant women receive an ultrasound (US) scan prior to 24 weeks gestation to encourage early identification of various conditions, such as fetal anomalies, multiple gestation, and placental abnormalities; however, global access to US remains limited. This has prompted many research groups to develop artificial intelligence (AI) approaches for obstetric US.

**Objective:**

The aim of this study was to update and synthesize current literature regarding the development of AI algorithms for obstetric US.

**Search Strategy:**

Methods were modified from Horgan et al. scoping review on the progress of AI algorithms for obstetric US. Our search, which encompassed papers published between 1991 and May 2022, adapted Horgan's search strategy by replicating the search strings across PubMed, Cochrane Library, and clinicaltrials.gov databases, while also snowballing additional references.

**Selection Criteria:**

Studies included both AI and obstetric US with a focus on one or more maternal and/or fetal conditions between January 2022 and January 2024. After removing duplicates, publications were screened for inclusion criteria based on their mention of both AI and obstetric US and a main objective assessing maternal and/or fetal conditions. Studies were excluded if they failed to mention the use of AI or obstetric ultrasound, discussed AI algorithm development, or consisted of expert opinions, reviews, and abstracts.

**Data Collection and Analysis:**

We used Zotero to manage references and extracted data onto an Excel template. The remaining publications were reviewed for data extraction including—authors, dates, objectives, settings, and funding sources. Publications were categorized into seven main areas based on Horgan's framework, with additional subcategories for emerging topics. Descriptive statistics summarized the data, with graphical visualizations depicting the geographic distribution of studies.

**Main Results:**

A total of 96 articles were included in the final results, revealing the rapid increase in the number of publications related to AI in obstetrics. The greatest proportion of studies were categorized as fetal biometry (25%) and anatomical evaluation of the fetus (20%). Studies took place across multiple regions with the greatest number in Asia (41%) and Europe (27%). A total of 22% were conducted in low‐ or middle‐income countries (LMICs).

**Conclusion:**

This scoping review demonstrates the growth and development of AI‐enabled obstetric US applications. There is a wide variety of innovative applications on the horizon and implementation approaches and implications should be explored as these technologies become clinically available. We encourage development of algorithms that focus on parameters that identify conditions linked to the global burden of maternal and neonatal mortality and morbidity, such as gestational age and placental location.

## 1 INTRODUCTION

In 2016, the WHO released global antenatal recommendations for a positive pregnancy experience.^1^ These guidelines recommend one obstetric ultrasound (US) scan for pregnant women prior to 24 weeks gestation for assessment of gestational age and early detection of conditions including congenital anomalies, multiple gestation, and placental abnormalities.^1^

Despite the emergence of hand‐held point‐of‐care ultrasound (POCUS), global US access remains limited due to a number of challenges. One of the biggest challenges is the lack of trained specialists to provide US. In high‐income countries, like the USA, the distribution of obstetrician and gynecologists (OBGYNs) correlates with population density, resulting in greater access to care in urban areas compared to rural regions where such specialized care is scarce.^2^ This pattern reflects a global trend where the availability of specialty‐trained medical professionals, including OBGYNs and radiologists, is inadequate, especially in resource‐limited settings.^3^ The lack of trained specialists hinders widespread implementation of routine obstetric US; however, efforts to train lower cadres of providers, such as midwives and nurses, have been effective.^1^ In low‐ and middle‐income countries (LMICs), in particular, task sharing has been shown to increase US access in primary health care settings and improve management of pregnant women, but issues involving staffing, workload, and infrastructure remain.^4^, ^5^

Recently, artificial intelligence (AI) has emerged as a new technology to potentially increase access to US. One example use case includes AI‐enabled POCUS that can be administered by non‐specialist providers at the bedside. In this approach, the provider conducts blind sweeps (sweeping the handheld probe across the pregnant abdomen several times in the instructed directions) to obtain images that are analyzed and interpreted through specialized algorithms to identify pregnancy complications.^6^ Although these AI algorithms are currently under development and not yet approved or widely implemented, it is envisioned that this new technology may expand routine access to US by shortening training duration, improving user guidance of the probe, and enhancing imaging pattern recognition.^7^, ^8^, ^9^ This may ultimately increase access by decreasing the reliance on trained sonographers or specialists.

In addition to this one potential use case, several other AI algorithms and applications are under development. A 2023 scoping review by Horgan et al. described the scope of AI applications for obstetric US prior to mid‐2022 including first trimester pregnancy US, assessment of placenta, fetal biometry, fetal echocardiography, fetal neurosonography, assessment of fetal anatomy, and other uses.^10^ The objective our scoping review was to document the evolving applications of AI‐enabled in obstetric US and identify funders and geographies where these efforts are underway. Understanding the tools that are on the horizon may identify gaps in existing use cases and prioritize potentially high‐impact applications. This has potential to improve maternal and neonatal outcomes globally that warrant development efforts.

## 2 METHODS

### 2.1 Study design

This scoping review highlights the recent development of AI algorithms for obstetric US between 2022 and January 2024. We adapted Horgan's search strategy by first replicating the search strings across PubMed, Cochrane Library, and clinicaltrials.gov databases, while also snowballing references to identify additional relevant sources not captured in the initial search. We adapted the inclusion and exclusion criteria and data extraction parameters to get a better understanding of current efforts and highlight the changing landscape.

### 2.2 Search strategy

We searched PubMed, Cochrane Library, and clinicaltrials.gov between 2022 and January 2024 using the search string in Horgan et al.: (“deep learning” OR “machine learning” OR “artificial intelligence” OR “neural networks”) AND (obstetrics OR obstetrical OR fetus OR foetus OR foetal OR fetal OR pregnancy OR pregnant) AND (ultrasound). Table 1 presents the keywords used for all databases.

**TABLE 1:** Search terms.

Artificial intelligence	Obstetrics	US
“Deep learning,” “machine learning,” “artificial intelligence,” “neural networks”	Obstetrics, obstetrical, fetus, foetus, foetal, fetal, pregnancy, pregnant	Ultrasound

Through this process we encountered and snowballed in relevant publications from 2014–2023 that were not captured in the scoping review by Horgan et al. of 127 publications between 1991–2022. Snowballed references included publications that were indexed outside of Horgan's databases, or did not contain key search terms in their title and abstract, such as publications that used “automated” to indicate AI algorithms. Throughout this process, we solicited guidance from the UCSF librarian to optimize and validate our search methodologies.

### 2.3 Selection of relevant studies

Studies were included based on their mention of both AI and obstetric US and whose main objective was the assessment of maternal and/or fetal conditions. We adapted the same definition of AI and obstetric US from the Horgan et al. publication for this criteria: “Obstetric US was defined as the process of obtaining US images of a fetus, amniotic fluid, or placenta and AI was defined as the use of neural networks, machine learning, or deep learning methods.”^10^ We did not apply quality criteria for inclusion and a formal quality appraisal was not conducted. However, all studies were assessed by two reviewers to ensure relevance and eligibility, as described below.

Studies were excluded based on several criteria. All expert opinions, reviews, and abstracts were removed, regardless of their mention of AI in obstetric US. Studies that did not explicitly mention the use of AI or obstetric US were also removed. Studies that developed AI algorithms that comprised standard US findings together with medical history or clinical factors were excluded.

### 2.4 Data extraction and analysis

All papers identified via PubMed, Cochrane Library and clinicaltrails.gov were entered into Zotero and exported to an excel template to assess inclusion and exclusion criteria. After removing duplicates, we conducted a title scan to remove articles that did not focus on AI or obstetric use. Throughout this screening process, authors VG and DW cross‐checked each other's work to confirm appropriate inclusion and exclusion of publications. Any discrepancies were discussed and resolved.

Data extraction from each of the publications included: author, publication date, title, main objective, setting, and funder. Based on the author‐stated main study objective(s), we categorized publications into seven categories that focused on the specific uses of AI in obstetric US following Horgan's categorization (Table 2): (1) Anatomical evaluation of the fetus, (2) fetal biometry, (3) fetal cardiac imaging, (4) fetal neurosonography, (5) first trimester US, (6) placenta US, and (7) other. After the main categorization, each publication was further placed into a subcategory, describing its specific use case. While the seven main categories were directly repurposed from Horgan et al., the subcategories were updated based on the emerging topics. The setting was extracted by scanning the title, abstract, or methods of each publication while the funder was identified for each publication under a designated “funding” or the “acknowledgments” sections.

**TABLE 2:** Description of obstetric categories for AI‐enabled US.

Category	Description
Anatomical evaluation of the fetus	Fetal anomalies
Fetal biometry	Fetal anatomy for gestational age
Fetal cardiac imaging	Structural, cardiac anomalies
Fetal neurosonography	Neurodevelopmental and structural anomalies
First trimester US	Sac measurement and crown rump length for gestational age assessment
Placenta US	Placental characteristics (location, thickness, etc.)
Other	Alternate uses of AI‐enabled obstetric US

Abbreviations: AI, artificial intelligence; US, ultrasound.

Categorization of each study was completed and cross‐checked by authors V.G. and DW Author VG conducted the initial review of titles and abstracts, followed by a second review from author DW for finalization of inclusion and exclusion of publications. The two‐step review process was repeated for data extraction. Inconsistencies were discussed until both authors were in agreement.

Data from included studies were summarized using descriptive statistics, primarily counts, and frequencies. Data were mapped using graphical visualizations to display geographic distribution of studies. In cases where a single study addressed multiple categories, the study was included in each relevant category and identified with an asterisk next to the author and year (e.g., total categorized studies exceeded 96 included publications).

### 2.5 Ethical considerations

IRB approval was not required as this scoping review did not include human subjects research.

## 3 RESULTS

**FIGURE 1:**
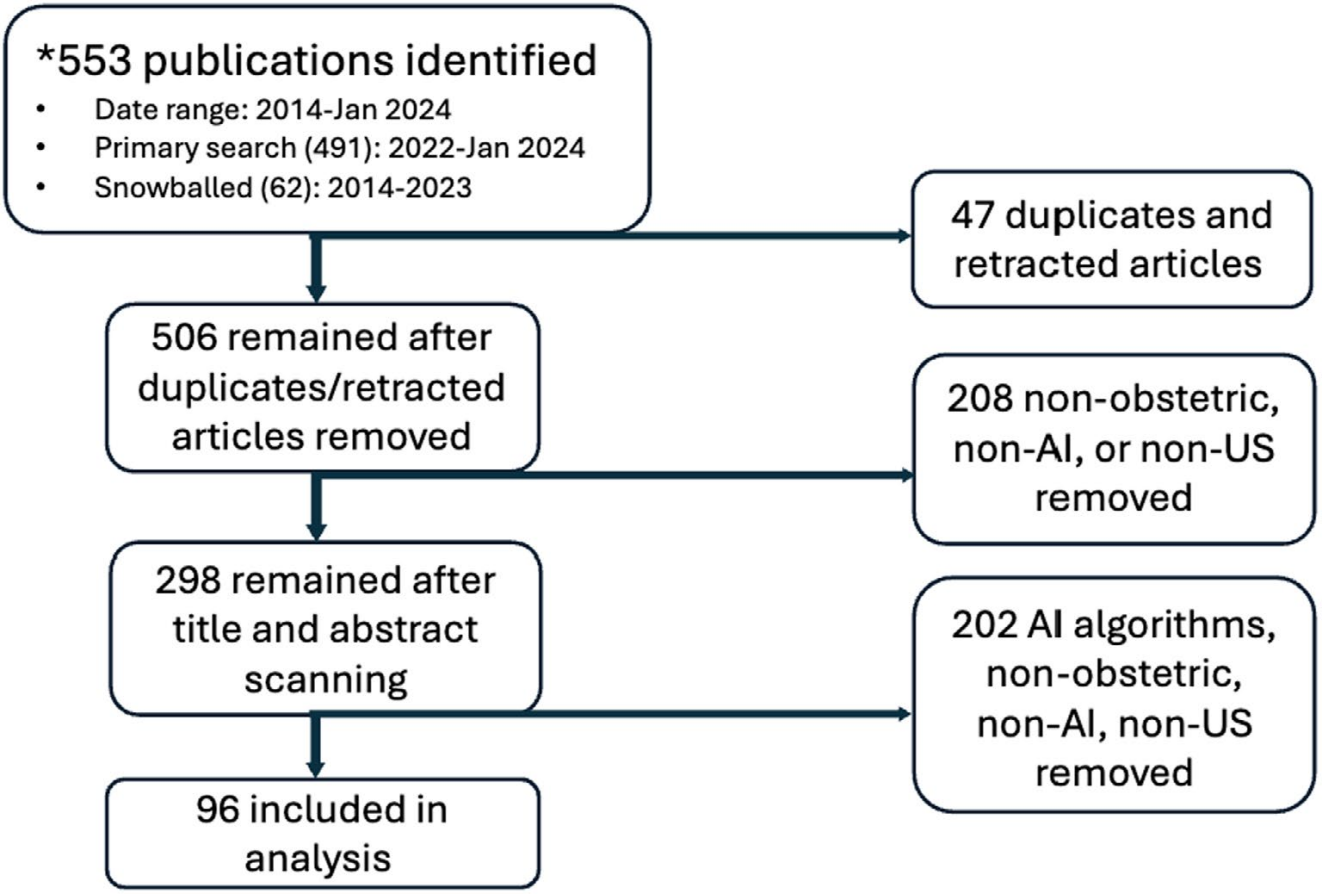
Flow chart of the studies identified and selected.

We identified 491 papers via PubMed between 2022–2024 and 24 studies were identified from both Cochrane Library and clinicaltrials.gov between 2014–2023. An additional 38 references were snowballed in published between 2014–2024, totaling 553 publications. Following a title scan, 208 were removed as they did not focus on AI or obstetric use. After review of the abstract, an additional 200 studies were excluded. A total of 96 studies fulfilled the inclusion criteria: 81 references were from PubMed, two were from Cochrane Library, two were from clinicaltrials.gov, and 11 were snowballed in Figure 1.

Table 3 shows the distribution of the seven categories of AI for obstetric US described in each study, where eight studies were relevant to more than one category. Fetal biometry‐related studies had the highest frequency of studies (25%, *n* = 26), followed by anatomical evaluation of the fetus (20%, *n* = 21). For the other categories: 13% (*n* = 14) were categorized under fetal cardiac imaging; 11% (*n* = 11) fetal neurosonography; 12% (*n* = 12) placenta US; 9% (*n* = 9) first trimester US; and 11% (*n* = 11) “other.”

**TABLE 3:** Summary of studies.^11^, ^12^, ^13^, ^14^, ^15^, ^16^, ^17^, ^18^, ^19^, ^20^, ^21^, ^22^, ^23^, ^24^, ^25^, ^26^, ^27^, ^28^, ^29^, ^30^, ^31^, ^32^, ^33^, ^34^, ^35^, ^36^, ^37^, ^38^, ^39^, ^40^, ^41^, ^42^, ^43^, ^44^, ^45^, ^46^, ^47^, ^48^, ^49^, ^50^, ^51^, ^52^, ^53^, ^54^, ^55^, ^56^, ^57^, ^58^, ^59^, ^60^, ^61^, ^62^, ^63^, ^64^, ^65^, ^66^, ^67^, ^68^, ^69^, ^70^, ^71^, ^72^, ^73^, ^74^, ^75^, ^76^, ^77^, ^78^, ^79^, ^80^, ^81^, ^82^, ^83^, ^84^, ^85^, ^86^, ^87^, ^88^, ^89^, ^90^, ^91^, ^92^, ^93^, ^94^, ^95^, ^96^, ^97^, ^98^, ^99^, ^100^, ^101^, ^102^, ^103^, ^104^, ^105^

Study categorization	Number of studies (*n* = 96^a^)
Anatomical evaluation of the fetus	21 (20%)
Classification of fetal structures	4
Detection of standard anatomic planes	10
Other	7
Fetal biometry	26 (25%)
Assessment of fetal brain morphology for estimation of GA	1
Detection of fetal abdominal standard planes	1
Femur length only	1
Fetal head measurements	6
Measurements of fetal biometry	1
Various combinations of ≥2 fetal biometry measurements	14
Other	2
Fetal cardiac imaging	14 (13%)
Automatic segmentation of the fetal heart and lungs	2
Assessment of the four‐chamber view	5
Detection of heart substructure to calculate abnormality score	1
Detection of standard fetal heart views (abnormal or normal)	4
Other	2
Fetal neurosonography	11 (11%)
Automated detection and measurement of ≥1 intracranial structure	2
Classification of fetal head biometry	3
Segmentation of planes and fetal brain	5
Other	1
First trimester US	9 (9%)
Detection of gestational sac	1
Fetal anatomy	3
Measurement of nuchal translucency	1
Standardization of planes	2
Other	2
Placenta US	12 (12%)
First trimester placental volume for the prediction of SGA neonates	1
Categorization of placental location	3
Comparison of placental texture throughout pregnancy in patients with hypertensive disorders to normotensive patients	3
Detection of placental location	1
Segmentation of the placenta only	1
Other	3
Other	11 (11%)
Assessment of amniotic fluid	1
Doppler FHR	2
Fetal descent	1
Gender blocking	1
Optimizing standard US	2
Preterm birth prediction cervical characteristics	2
User accuracy	2

Abbreviations: FHR, fetal heart rate; GA, gestational age; SGA, small‐for‐gestational age; US, ultrasound.

^a^ Studies listed in more than one category.

Table 4 compares the categorization differences between Horgan et al. and the current search. Across the two time periods, the proportion of studies that focus on anatomical evaluation of the fetus increased from 5% to 20%. There is also a decrease in the proportion of studies focusing on fetal biometry (37%–25%). Overall, there has been a substantial increase in the volume of publications; the Horgan et al. scoping review spanned 31 years, while this updated scoping review covered only 2 years.

**TABLE 4:** Horgan versus updated search.

Study type	Horgan (1991–2022)	Updated search (2022–2024)
Anatomical evaluation of the fetus	6 (5%)	21 (20%)
Fetal biometry	47 (37%)	26 (25%)
Fetal cardiac imaging	10 (8%)	14 (13%)
Fetal neurosonography	20 (15%)	11 (11%)
First trimester US	11 (9%)	9 (9%)
Placenta US	8 (6%)	12 (11%)
Other	25 (20%)	11 (11%)
Total number of included studies	127	104

Abbreviation: US, ultrasound.

**FIGURE 2:**
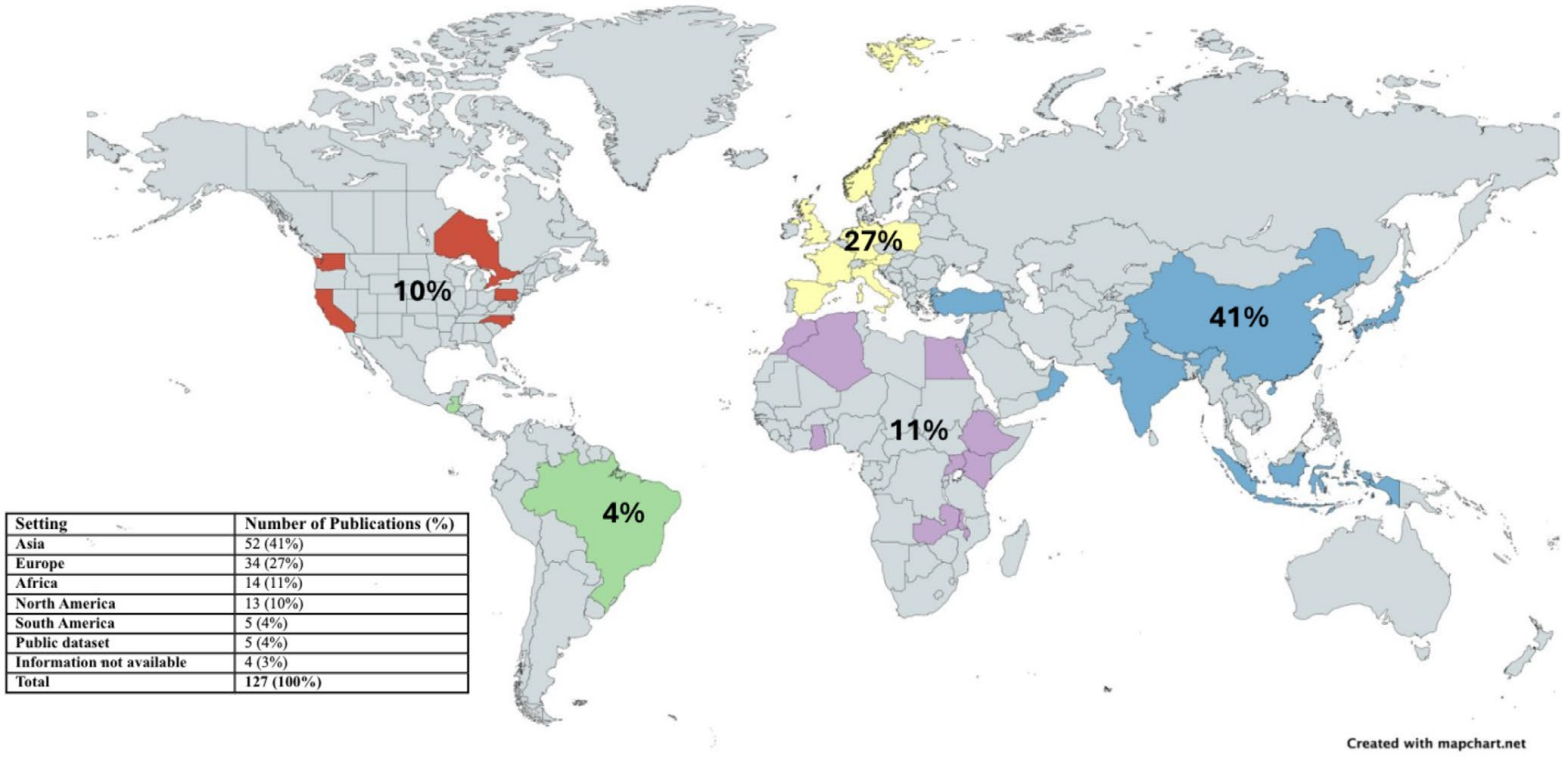
Regions of artificial intelligence (AI)‐enabled obstetric ultrasound (US) efforts: Percentages represent the number of studies conducted in those regions.

Recent studies related to AI‐enabled obstetric US span various geographic settings (Figure 2).^106^ Specific countries where each study took place are highlighted. A total of 20 studies took place across multiple regions, and therefore were counted multiple times, resulting in a total *n* = 127 (Tables S1–S7). A total of 3% (*n* = 4) of studies did not specify a location while 4% (*n* = 5) of studies leveraged public datasets with no region specified. Asia (blue) had the highest proportion of studies 41% (*n* = 52), followed by Europe with 27% (*n* = 34). Africa had 11% (*n* = 14) studies that took place across multiple countries. North America had 10% (*n* = 13) that took place across multiple countries and US states. Central and South America had the lowest number of studies conducted (4%, *n* = 5). A total of 22% (*n* = 28) of the 127 regions mentioned took place in LMICs.

Funding for this work was varied; across the 96 studies, five types of funders were identified (Table 5). There were 207 funders for the 96 studies reflecting the fact that many studies had more than one funder. The primary funders were government agencies (53%), followed by academic institutions (20%). Foundations, industry and private companies funded 16%, 4% and 4% of studies, respectively. Some studies did not report the funding source (8%).

**TABLE 5:** AI‐enabled obstetric US study funding source.

Funders	Number of funders (%)
Government	109 (53%)
Academic	42 (20%)
Foundation	16 (8%)
Information not available	16 (8%)
Industry	9 (4%)
Private	9 (4%)
No external funding	6 (3%)
Total	207

Abbreviations: AI, artificial intelligence; US, ultrasound.

## 4 DISCUSSION

This scoping review of AI for obstetric ultrasound reveals the growing interest and potential for this promising technology. Our findings reflect a rapid expansion in the number and diversity of AI algorithms under development for obstetric use.

The number of studies related to AI algorithm development for obstetric US use has increased significantly in recent years. A comparison between the Horgan et al. scoping review from 1991 to May 2022, and this updated search which included January 2022–January 2024, reveals an increase in the proportion of publications focused on the anatomical evaluation of the fetus, fetal cardiac imaging, and placenta US, fetal biometry, and fetal neurosonography. Over a 31‐year period, 127 studies described applications of AI‐enabled obstetric US. In contrast, this updated search covering only a two‐year period identified 96 publications. Furthermore, a quick search using the same criteria between February 2024 and April 2024, revealed an additional 100 studies in just four months. While these additional 100 studies are not described in this scoping review, this observation underscores just how rapidly this field is changing.

The rapid expansion of AI applications for obstetric US has important implications that must be considered. It reflects a high degree of innovation which is favorable at these early stages. However, we found little evidence of coordination and collaboration across studies and regions. Coordinating future research may help synchronize findings as AI continues to progress, as well as offer opportunities to share and diversify datasets used in AI algorithm development. Shared collaboration may enhance algorithmic robustness geographic representation, and population‐specific diversity. For example, AI algorithms need to consider that fetuses develop differently across settings. Some regions in the Global South experience higher rates of small‐for‐gestational age or intrauterine growth restriction than those in the Global North.^107^ If the majority of studies to train algorithms are being conducted in a single population or geographic region, this raises the question of how generalizable the algorithm will be when assessing maternal and fetal conditions, including measures related to fetal biometry or growth.^108^ This also emphasizes the need for high‐quality validation studies and quality assurance checks before clinical implementation can occur.^9^

This review illustrates the growing diversity of applications for AI in obstetric US. For example, the emergence of AI applications for Doppler assessments of umbilical flow, optimization of standard US measure, and enhancing user accuracy necessitated adaptation of the subcategories originally listed in the review by Horgan et al. Another innovation for AI in obstetric US is to enhance traditional US techniques, thus improving the quality and accuracy of standard US. We also found four studies on general data augmentation (creating new data from existing data for machine learning models) for obstetric US during our initial search; however, these were excluded because they did not assess a specific fetal or maternal condition.

Recognizing that funders play a role in driving innovation is important. Our analysis of the various funding agencies/sponsors involved in this work underscores not only the growing interest in this field, but also an opportunity to enhance collaboration and coordination. This scoping review revealed governmental organizations as the primary funders, with academic institutions following. Given recent changes in the fundings ecosystem, the influence funding sources have on future AI development priorities may warrant exploration. Similarly, the role of funders ensuring best practices and safeguards in the generation of AI‐enabled tools remains to be defined.^109^

The primary strength of this scoping review is a detailed description of the rapidly evolving landscape of AI‐enabled obstetric US. It is important to address the limitations of this scoping review. While our search string did include “pregnancy” and “pregnant” which encapsulated some publications discussing maternal conditions, the search string did not include the word “maternal.” We did not change Horgan's initial search string in order to accurately compare the growth in the field since their scoping review. However, a search on PubMed including “maternal” in the original search string yielded 137 studies between 1991–2024. Though this observation does not assess relevance, it may suggest some studies may be missing from this scoping review. Thus, there is opportunity for future reviews and categorization efforts to ensure the focus is on the mother in addition to the fetus. Additionally, although we included all studies that met the inclusion criteria through a two‐step review process, a formal quality appraisal of each study was not conducted.

This literature review focused on the development of AI‐enabled applications for obstetric US. Though outside the scope of this review, we did not identify any publications describing implementation experiences or clinical use of any existing AI‐enabled US tools. As these algorithms and applications progress beyond the R&D phase, it will be important for future efforts to consider implementation factors like user friendliness, cost effectiveness, and compatibility with existing infrastructure to smoothly integrate this technology into standard clinical practice. Ensuring that future AI‐enabled devices are designed with implementation in mind is critical.^110^

## 5 CONCLUSION

The rapid acceleration in AI algorithm development for obstetric reflects the interest and investment in this promising technology. Efforts to collectively prioritize and coordinate research and innovation in this field are recommended. Similarly, as these technologies become available in clinical practice globally, it is important to encourage innovation and adoption that is driven by local priorities to address the health needs of pregnant people, newborns, and the providers that care for them.

## AUTHOR CONTRIBUTIONS

All authors contributed to the conception and design of this study. VG and SDR led data collection. VG and DW were involved in data analysis and interpretation. VG drafted the manuscript and all authors revised the manuscript and gave their final approval for the submitted version.

## FUNDING INFORMATION

This work was performed by the first author as a thesis project and was included as a component of a contract from the Bill and Melinda Gates Foundation.

## CONFLICT OF INTEREST STATEMENT

The authors have no conflicts of interest.

## Supporting information


**Appendix S1:** Supporting Information.

**Appendix S2:** Supporting Information.

## Data Availability

The data that support the findings of this study are available from the corresponding author upon reasonable request.
