# Supplementary material for: Emerging applications of artificial intelligence for obstetric ultrasound: A scoping review
Source: Int J Gynaecol Obstet. 2026 Jan 9;174(1):34–43. doi: 10.1002/ijgo.70789 (PMC13278657; doi:10.1002/ijgo.70789)
Supplement: Supplementary file 2 — Appendix S2: Supporting Information. [file IJGO-174-34-s001.docx]

**SUPPLEMENTARY FILES**

**Anatomical Evaluation of the Fetus**

21 studies (20%) addressed anatomical evaluation of the fetus. Four of these studies focused on the classification of fetal structures. Ten of these studies focused on the detection of standard anatomic places, and seven studies fall under “other.” Of these 21 studies, about half of them took place in both Asia (n=10) and Europe (n=9). Table S1 further summarizes the publications in this category.

TABLE S1. Anatomical evaluation of the fetus

| **Author, Year** | **Subcategorization** | **Objective** | **Setting** | **Funding** |
| --- | --- | --- | --- | --- |
| Anto, 2015 | Classification of fetal structures | To segment head contour in fetal US scans acquired in low-cost settings, such as acquisition performed in rural areas of low-income countries using portable machines. | Biriwa, Ghana | Information not available |
| *Gomes, 2022 |  | To investigate the use of AI for fetal US in under-resourced settings. | Chapel Hill, North Carolina, United States and Lusaka, Zambia | Foundation |
| Lei, 2023 |  | To develop and validate a prenatal-screening artificial intelligence system (PSAIS) for real-time evaluation of the quality of anatomical images, indicating existing and missing structures. | Dataset from Guangzhou, China | Academic (2)  Government (3) |
| Kaplan, 2022 |  | To develop an efficient automated US-based fetal sex classification model that can facilitate efficient screening and reduce misclassification. | Malatya, Turkey | Information not available |
| Lu, 2022 | Detection of standard anatomic planes | To present a multitask network with a shared feature encoder and three task-special decoders for standard plane recognition, image segmentation of pubic symphysis (PS) and fetal head, and endpoint detection of PS. | Guangzhou, China | Academic  Government (4) |
| Guo, 2023 |  | To improve the acquisition efficiency and accuracy of the US standard plane, using a novel detection framework that utilizes both the coarse-to-fine detection strategy and multi-task learning mechanism for feature-fused images. | Shenzhen, China | Government (2) |
| Rahman, 2023 |  | To identify fetal planes from US images with higher precision. | Dataset from Barcelona, Spain | Academic |
| Sendra-Balcells, 2023 |  | To investigate different strategies to reduce the domain-shift effect arising from a fetal plane classification model trained on one clinical center with high-resource settings and transferred to a new center with low-resource settings. | Egypt, Algeria, Uganda, Ghana and Malawi | Academic  Government (2)  Foundation |
| Dapueto, 2024 |  | To recognize automatically standard planes in fetal US images with an efficient procedure, applicable onboard of portable US device, with limited computational power. | Genova, Italy | Academic |
| *Pei, 2023 |  | To develop and validate a fully automated AI system to extract standard planes, assess early gestational weeks, and compare the performance of the developed system to sonographers. | Guangzhou, Zhujiang New Town, and Zengcheng, China | Academic |
| Tang, 2023 |  | To develop a three-stage model that utilizes fetal US images to detect genetic disorders. | Guangzhou, China | Academic  Government |
| Gao, 2023 |  | To propose a complete US fetal examination system to deal with this troublesome problem by repairing and screening the anatomically implausible results. | GE Medical Systems dataset geographic setting unavailable; Zipf, Austria; Barcelona, Spain | Government |
| Lasala, 2023 |  | To generate synthetic fetal head standard planes (FHSP) images with conditional generative adversarial network (cGAN) using class activation maps (CAMs). | Dataset from Barcelona, Spain | Private |
| Lee, 2023 |  | To estimate gestational age using only image analysis of standard US planes, without any measurement information via machine learning methods. | Brazil; China; India; Italy, Kenya; Oman; UK; USA | Academic  Foundation |
| Clinicaltrials, 2022 | Other | To detect fetal anomalies between women with a fetus diagnosed with a structural malformation and control groups. | London, England | Government |
| Tang, 2023 |  | To effectively screen genetic diseases of the fetus early via a noninvasive screening tool. | Guangzhou, China | Academic  Government (3) |
| Tang, 2023 |  | To develop a fully automated prenatal screening algorithm (Pgds-ResNet) based on deep neural networks that detects high-risk fetuses affected by a variety of genetic diseases. | Guangzhou, China | Academic  Government (3) |
| Ghabri, 2023 |  | To automate fetal organ classification from US images via deep learning models. | Barcelona Spain; Egypt; Algeria; Uganda; Malawi; Ghana | Academic (2) |
| Zhang, 2022 |  | To develop and validate a deep learning model for screening fetuses with trisomy 21 based on ultrasonographic images. | Beijing and Shijiazhuang, China | Information not available |
| Plotka, 2023 |  | To develop and test a deep learning method to automatically estimate fetal weight from fetal abdominal US video scans. | Warsaw, Poland | Government (3) |
| Plotka, 2023 |  | To automatically predict fetal birth weight by using fetal US video scans and clinical data. | Warsaw and Poznan, Poland | Government (2)  Industry |

*overlap across multiple categories (*); all objectives were directly adapted from each publication to prevent the original aim of the paper from being misconstrued; # in () reflects number of unique funders*

**Fetal Biometry**

26 papers (25%) addressed fetal biometry (Table 3). Of the 26, one focused on the assessment of fetal brain morphology for estimation of gestational age. Another study focused on the detection of fetal abdominal standard planes. One study focused on femur length only. Six studies focused on fetal head measurements. One study focused on measurements of fetal biometry. Two studies fall under “other”. 14 studies involved various combinations of two or more fetal biometry measurements. Of these 26 studies, 8 took place in Asia, 10 took place in Europe, 7 took place in Africa, 6 took place in North America. None took place in South America. Two of these studies are from publicly available datasets where the setting is not specified. Table S2 further summarizes the publications in this category.

TABLE S2. Fetal Biometry

| **Author, Year** | **Subcategorization** | **Objective** | **Setting** | **Funding** |
| --- | --- | --- | --- | --- |
| Dan, 2023 | Assessment of fetal brain morphology for estimation of GA | To develop an automatic DeepGA model to achieve fully automatic GA prediction in an end-to-end manner. | Guangzhou and Foshan, China | Academic  Government (4) |
| Khan, 2016 | Detection of fetal abdominal standard plane | To develop an automatic method for localization of the presented section through the abdomen and measurement of mean abdominal diameter (MAD). | Trondheim, Norway | Industry |
| Khan, 2015 | Femur length only | To develop an automatic method of detecting and measuring fetal femur length that can run on a tablet device to assist the health care worker during the scanning process. | Trondheim, Norway | Information not available |
| Van Den Heuvel, 2018 | Fetal head measurement | To present a computer aided detection (CAD) system for automated measurement of the fetal HC in 2D US images for all trimesters of the pregnancy. | Nijmegen, Netherlands | Government |
| Yang, 2022 |  | To propose a fast and accurate method for fetal head circumference auto-measurement. | China | Academic  Government (3) |
| Pokaprakarn, 2022 |  | To devise an automated approach for gestational age assessment through US imaging. | Lusaka, Zambia; Chapel Hill, North Carolina, USA | Foundation |
| Zeng, 2022 |  | To devise an efficient method for automated fetal head circumference measurement from US images, optimizing for speed and suitability on resource-constrained devices. | Public dataset from Nijmegen, the Netherlands | Government (2) |
| Wang, 2022 |  | To introduce a novel convolutional neural network (CNN) called GAC Net to address challenges in US fetal head edge detection. | Public HC18 US fetal HC automatic measurement dataset, geographic setting unavailable. | No financial relationship with other people or organizations |
| Devisri, 2023 |  | To propose a fetal growth analysis through US image of head circumference biometry using optimal segmentation and hybrid classifier. | Open benchmark datasets HC18 geographic setting unavailable; Oxford University | Information not available |
| Pokaprakarn, 2021 | Measurement of fetal biometry | To estimate gestational age from blind US sweeps in low-resource settings using deep learning algorithms | Lusaka, Zambia; Chapel Hill, North Carolina, USA | Foundation |
| Teng, 2022 | Other | To investigate the effect of nuchal fold (NF) in predicting fetal growth restriction (FGR) using machine learning. | Kent Ridge, Singapore | Information not available |
| Gao, 2023 |  | We aimed to develop an accurate and widely applicable screening model for SGA at 21-24 gestational weeks of singleton pregnancies. | Shanghai, China | Government (2) |
| Ambroise, 2019 | Various combinations of ≥2 fetal biometry measurements | 1. To assess the consistency between 3D measurements (automated and manual) extracted from a fetal US volume with standard 2D US measurements.  2. To evaluate the feasibility of the use of software to obtain automated measurements of the fetal head, abdomen and femur from US acquisitions  3. To assess the impact of automation on intraobserver and interobserver reproducibility. | France | Industry |
| *Self, 2022 |  | To develop Computer-Assisted Low-Cost Point-of-Care Ultra-Sound (CALOPUS) for nonexpert users to assess fetal viability, detect the presence of multiple pregnancies, evaluate placental location, assess amniotic fluid volume, determine fetal presentation, and perform basic fetal biometry. | UK; India | Government |
| Jatmiko, 2015 |  | 1. To develop an integrated automatic system for fetal growth monitoring and detection, encompassing automated measurement of fetal head, abdomen, femur, and humerus parameters.  2. To integrate telehealth monitoring system to enhance US accessibility for rural patients.  3. To propose a new approach of fetal image detection using AdaBoost. | Indonesia | Academic |
| *Pei, 2023 |  | To develop and validate a fully automated AI system to extract standard planes, assess early gestational weeks, and compare the performance of the developed system to sonographers. | Guangzhou, Zhujiang New Town, and Zengcheng, China | Academic |
| Liu, 2021 |  | To develop an end-to-end network facilitating automated measurements of fetal HC and fetal abdomen circumference from 2D US images across pregnancy trimesters. | China | Information not available |
| Alzubaidi, 2022 |  | To develop a full end-to-end framework for segmenting, measuring, and estimating fetal gestational age and weight based on two-dimensional US images of the fetal head. | Nijmegen, Netherlands | Government |
| Slimani, 2023 |  | To develop and assess the performance of deep learning models for end-to-end automation of fetal biometry and amniotic fluid volume measurements. | Morocco | Industry |
| Lee, 2023 |  | To develop AI models to estimate GA with higher accuracy and reliability, leveraging standard biometry images and fly-to ultrasonography videos. | Lusaka, Zambia; Chapel Hill, North Carolina, USA | Foundation  Industry |
| Rittenhouse, 2023 |  | To assess he accuracy of two portable US machines (PUM) in obtaining fetal biometry and estimating gestational age. | Lusaka, Zambia; Chapel Hill, North Carolina, USA | Information not available |
| Viswanathan, 2024 |  | To create a tool to reduce substandard fetal biometry measurement while minimizing care disruption. | Lusaka, Zambia; Chapel Hill, North Carolina, USA | Foundation |
| *Sarker, 2023 |  | To classify maternal-fetal and brain anatomical structures present in 2-D fetal US images using a deep-learning-based image classification architecture called the COMFormer | Dataset from Barcelona, Spain | Information not available |
| *Gomes, 2022 |  | To investigate the use of AI for fetal US in under-resourced settings. | Lusaka, Zambia; Chapel Hill, North Carolina, USA | Foundation |
| Gabler, 2023 |  | To evaluate the feasibility of fetal re-identification on FETAL_PLANES_DB, a publicly available dataset of singleton pregnancy US images. | Dataset from Barcelona, Spain | Government |
| Gembicki, 2023 |  | 1. To evaluate the accuracy and efficacy of AI-assisted biometric measurements of the fetal CNS by comparing two semiautomatic postprocessing tools.  2. To discuss the additional value of semiautomatically generated sagittal and coronal planes of the CNS. | Lübeck, Germany | This research received no external funding |

*gestational age (GA), head circumference (HC), abdomen circumference (AC); overlap across multiple categories (*); all objectives were directly adapted from each publication to prevent the original aim of the paper from being misconstrued; ; # in () reflects number of unique funders*

**Fetal Cardiac Imaging**

14 papers (13%) addressed fetal cardiac imaging (Table 3). Of the 14 papers, two studies focused on the automatic segmentation of the fetal heart and lungs. Five studies focused on the assessment of the four-chamber view. One study focused on the detection of heart substructure to calculate abnormality score. Four studies focused on the detection of standard fetal heart views as abnormal or normal. Two papers fall under the “other’ category. One paper focuses on the detection of the heart substructure to calculate abnormality score (AS). Of these 14 studies, the majority of them took place in Asia (n=10), followed by Europe (n=3). One study did not mention the setting of their study. Table S3 further summarizes the publications in this category.

TABLE S3. Fetal Cardiac Imaging

| **Author, Year** | **Subcategorization** | **Objective** | **Setting** | **Funding** |
| --- | --- | --- | --- | --- |
| Alkhodari, 2022 | Automatic segmentation of the fetal heart and lungs | To propose deep coherence, a novel AI approach that relies on 1 min non-invasive electrocardiography (ECG) to explain the association between maternal and fetal heartbeats during pregnancy. | Washington, United States;  Sendai and Kanagawa, Japan | Academic (2) |
| Wang, 2022 |  | To explore whether the post-left atrium space (PLAS) ratio would be useful for prenatal diagnosis of total anomalous pulmonary venous connection (TAPVC) using echocardiography and artificial intelligence. | Beijing, China | Academic (3)  Government |
| Veronese, 2023 | Assessment of the four chamber view | To test the fetal intelligent navigation echocardiography (FINE) method for prenatal diagnosis in a series of fetal atrioventricular septal defects (AVSD) patients. | Padua, Italy, | This research received no external funding. |
| Day, 2023 |  | To analyze the performance of the current national screening programme in detecting hypoplastic left heart syndrome (HLHS) to compare with our own AI model. | London, England | Academic  Foundation  Government |
| Du, 2022 |  | To develop a novel method for predicting neonatal respiratory morbidity (NRM) by US-based radiomics technology. | Shanghai, China | Government (3) |
| Lu, 2022 |  | To propose a YOLOX-based deep instance segmentation neural network (i.e., IS-YOLOX) for cardiac anatomical structure location and segmentation in fetal US images based on a multi-task deep learning framework. | Shenzhen, China | Government |
| Qiao, 2023 |  | 1. To propose a Pseudo-Siamese Feature Fusion Generative Adversarial Network (PSFFGAN), synthesizing high-quality fetal FC views using FC sketch images.  2. To propose a novel Triplet Generative Adversarial Loss Function (TGALF), which optimizes PSFFGAN to fully extract the cardiac anatomical structure information provided by FC sketch images to synthesize the corresponding fetal FC views with speckle noises, artifacts, and other ultrasonic characteristics. | Qingdao, China | Government (2)  Private |
| Nurmaini, 2022 | Detection of heart substructure to calculate AS | To analyze potential deep learning (DL) techniques to diagnose congenital heart disease (CHD) in fetal USs. | Palembang, Indonesia | Academic |
| Li, 2024 | Detection of standard fetal heart views as normal or abnormal | To develop a deep learning-based fetal heart US standard planes (FHUSPs) recognition network (FHUSP-NET) for automated recognition of FHUSPs and detection of key anatomical structures, for rapid and accurate prenatal US screening. | Fujian, China | Government (2) |
| Nurmaini, 2023 |  | To develop a stacked residual-dense network model to segment the entire region of cardiac and classifying their defect positions to generate automatic echocardiographic interpretation. | Palembang, Indonesia | Academic  Government |
| Wu, 2023 |  | To develop an effective AI recognition model is established by combining US images with artificial intelligence technology to assist US doctors in prenatal US fetal heart standard section recognition. | Fujian, China | Government (2) |
| Yang, 2023 |  | To compare different algorithm types utilized in assisting prenatal diagnosis of CHDs, using AI for enhanced diagnostic accuracy. | Information not mentioned | This publication received no external funding. |
| Pietrolucci, 2023 | Other | To evaluate the agreement between visual and automatic methods in assessing the adequacy of fetal cardiac views obtained during second trimester ultrasonographic examination. | Italy (to confirm) | Information not available |
| Tang, 2023 |  | To utilize the aortic arch view to screen for part of critical duct-dependent CHDs, including IAA, CoA and TGA. | Guangzhou, Shenzhen, and Guangdong, China | Academic  Government (4) |

*CHD = congenital heart disease; overlap across multiple categories (*); all objectives were directly adapted from each publication to prevent the original aim of the paper from being misconstrued; ; # in () reflects number of unique funders*

**Fetal Neurosonography**

11 studies (11%) address fetal neurosonography. Of these 11 studies, two focus on the automatic detection and measurement of more than one intracranial structure. Two studies focus on the classification of fetal head biometry. Six studies focus on the segmentation and planes of the fetal brain. One paper falls under the “other’ category. Of these nine studies, half of them took place in Asia (n=4) and Europe (n=4). Two studies received their data from an outside database. Three studies did not mention the setting of their study. One study was collected data from a public database where the setting was not mentioned. Table S4 further summarizes the publications in this category.

TABLE S4. Fetal Neurosonography

| **Author, Year** | **Subcategorization** | **Objective** | **Setting** | **Funding** |
| --- | --- | --- | --- | --- |
| Lin, 2023 | Automatic detection and measurement of ≥1 intracranial structure | To evaluate the efficacy of Prenatal US Diagnosis Artificial Intelligence Conduct System  (PAICS) in assisting fetal intracranial malformation diagnosis and compares the auxiliary diagnosis methods for the system. | Guangzhou and Xiamen, China | Government |
| Coronado-Gutiérrez, 2023 |  | To develop a novel pipeline using state-of-the-art deep learning methods to automatically delineate and measure several of the most important fetal brain structures assessed during mid-trimester routine fetal brain US examination | Dataset from Barcelona, Spain; Dataset from Fetal Planes DB, geographic setting unavailable. | Foundation (2) |
| Naiad, 2014 | Classification of fetal head biometry | To develop a technique for automatized computation of biparietal diameter (BPD) which can run on tablet devices. | Information not mentioned | Information not available |
| Maraci, 2020 |  | To develop a real-time image analysis framework for automatic detection and measurement of the transcerebellar diameter (TCD) in fetal brain US videos, resulting in accurate estimation of fetal GA. | Information not mentioned | Academic  Foundation  Government (5) |
| Alzubaidi, 2023 |  | To compile a dataset of 3832 high-resolution US images of fetal heads for applications in prenatal diagnostics, clinical diagnosis, and computer-assisted interventions. | Nijmegen, Netherlands; Dataset from Barcelona, Spain | Government |
| Lin, 2022 | Segmentation and planes of the fetal brain | To evaluate the efficacy of PAICS in assisting fetal intracranial malformation diagnosis and compares the auxiliary diagnosis methods for the system. | Guangzhou and Xiamen, China | Government |
| *Sarker, 2023 |  | To facilitate this objective in an automated fashion, we propose a deep-learning-based image classification architecture called the COMFormer to classify maternal–fetal and brain anatomical structures present in 2-D fetal US images. | Dataset from Barcelona, Spain | Government |
| Rauf, 2023 |  | To address the persistent gap in maternal mortality rates between low- and high-income countries, an automated computer-aided diagnostic (CAD) system using a novel deep learning architecture based on a residual bottleneck mechanism, to facilitate the identification of common maternal fetuses from US images. | Public dataset, geographic setting unavailable. | Academic |
| Moser, 2022 |  | To propose a convolutional neural network (CNN) that accurately and consistently aligns and extracts the fetal brain from minimally pre-processed 3D US scans. | Pelotas, Brazil; Turin, Italy; Muscat, Oman; Oxford, UK; Seattle, WA, USA; Beijing, China; Maharashtra, India; and Nairobi, Kenya | Academic (3)  Foundation  Government (2)  Private |
| Wright, 2023 |  | To propose a novel method to fuse the partially imaged fetal head anatomy, acquired from numerous views, into a single coherent 3D volume of the full anatomy. | Information not mentioned | Academic  Foundation (2)  Government |
| Miyagi, 2023 | Other | To examine whether AI can achieve discoveries regarding fetal brain activity. | Okayama, Japan | Information not available |

*overlap across multiple categories (*); all objectives were directly adapted from each publication to prevent the original aim of the paper from being misconstrue; ; # in () reflects number of unique funders*

**First Trimester US**

Nine studies (9%) address first trimester US. Of these nine studies, one paper focuses on the detection of the gestational sac. Three papers focus on first trimester fetal anatomy. One paper focuses on the measurement of nuchal translucency. Two of these papers focus on standard planes, and the remaining two are categorized under “other”. Of these nine studies, over half of them took place in Asia (n=7). Table S5 further summarizes the publications in this category.

TABLE S5. First Trimester US

| **Author, Year** | **Subcategorization** | **Objective** | **Setting** | **Funding** |
| --- | --- | --- | --- | --- |
| Wang, 2022 | Detection of gestational sac | To investigate the role of the convolutional neural network (CNN) in the prediction of spontaneous miscarriage risk through the analysis of early US gestational sac images. | Database from Liaoning, China | Academic  Government (2) |
| Gofer, 2022 | Fetal Anatomy | To evaluate the feasibility of machine learning (ML) tools for segmenting and classifying first-trimester fetal brain US images. | Tel Aviv, Israel | This study received no external funding. |
| Ji, 2023 |  | To study the validity of an AI model for measuring fetal facial profile markers, and to evaluate the clinical value of the AI model for identifying fetal abnormalities during the first trimester. | Suzhou, China | Academic  Private (3) |
| Walker, 2022 |  | To develop and internally validate a deep-learning algorithm from fetal US images for the diagnosis of cystic hygromas in the first trimester. | Ottawa, Canada | Government |
| Sun, 2021 | Measurement of nuchal translucency | To develop and validate a nomogram based on fetal nuchal translucency thickness (NT) and ultrasonographic facial markers for screening for trisomy 21 in the first trimester of pregnancy. | Beijing, China | Government (4)  Industry (2) |
| *Self, 2022 | Other | To develop Computer-Assisted Low-Cost Point-of-Care Ultra-Sound (CALOPUS) for nonexpert users to assess fetal viability, detect the presence of multiple pregnancies, evaluate placental location, assess amniotic fluid volume, determine fetal presentation, and perform basic fetal biometry. | UK; India | Government |
| Yasrab, 2023 |  | To examine imaging proficiency and practices of first trimester US scanning through analysis of full-length US video scans. | Oxford, UK | Government (3)  Industry |
| Zhen, 2023 | Standard planes | To develop a first-trimester standard plane detection (FTSPD) system that can automatically locate nine standard planes in US videos and investigating its utility in clinical practice. | Guangdong Province, China | Academic  Government  Private |
| *Pei, 2023 |  | To develop and validate a fully automated AI system to extract standard planes, assess early gestational weeks, and compare the performance of the developed system to sonographers; an optimal segmentation model was selected to outline gestational sacs. | Guangzhou, Zhujiang New Town, and Zengcheng, China | Academic |

*convolutional neural network (CNN), machine learning (ML); overlap across multiple categories (*); all objectives were directly adapted from each publication to prevent the original aim of the paper from being misconstrued*

**Placenta US**

12 studies (12%) address placenta US. Three studies focus on the categorization of placental location. Three studies focus on the comparison of placental texture throughout pregnancy in patients with hypertensive disorders compared to normotensive disorders. One paper focuses on the detection of placental location. One paper focuses on the segmentation of the placenta only. One paper focuses on the first trimester placenta volume for the prediction of SGA neonates. Three papers fall under the “other’ category and must be recategorized. Of these 12 studies, over half of them took place in Asia (n=7), followed by Europe (n=3). Table 11 further summarizes the publications in this category.

TABLE S6. Placenta US

| **Author, Year** | **Subcategorization** | **Objective** | **Setting** | **Funding** |
| --- | --- | --- | --- | --- |
| Schilpzand, 2022 | Categorization of placental location | To illustrate the feasibility of using automated placenta localization in a resource-limited setting. | Wolisso, Ethiopia | This study did not receive any outside funding. |
| *Self, 2022 |  | To develop Computer-Assisted Low-Cost Point-of-Care Ultra-Sound (CALOPUS) for nonexpert users to assess fetal viability, detect the presence of multiple pregnancies, evaluate placental location, assess amniotic fluid volume, determine fetal presentation, and perform basic fetal biometry. | UK; India | Government |
| Yang, 2022 |  | To explore the value of the deep dictionary learning algorithm in constructing a B US scoring system and exploring its application in the clinical diagnosis and treatment of pernicious placenta previa (PPP). | China | Government |
| Arora, 2023 | Comparison of placental texture throughout pregnancy in patients with hypertensive disorders to normotensive patients | To perform placental US image texture (UPIA) in first (T1), second (T2) and third(T3) trimesters of pregnancy using machine learning. | India | Government |
| Gupta, 2022 |  | To compare the placental quantitative US image texture of women with hypertension disorders of pregnancy (HDP) to those with the normal outcome. | India | Government |
| Sun, 2023 |  | To analyze and compare the placental features between normal and HDP pregnancies and propose a multimodal fusion deep learning model for differentiating and characterizing the placental features from HDP to normal pregnancy | Shanghai, China | Academic  Government |
| Gleed, 2023 | Detection of placental location | To develop a new method that automatically generates an assistive video overlay to provide image guidance to a user to assess placenta location. | Oxford, UK | Academic (2) |
| Schwartz, 2022 | First trimester placenta volume for the prediction of SGA neonates | To devise a novel convolutional neural network (CNN) pipeline for fully automated placenta segmentation from 3DUS images, exploring the association between the calculated PV and SGA. | Philadelphia, Pennsylvania, USA | Government (2)  Industry |
| Andreasen, 2023 | Other | To develop a deep learning-based model for classification and segmentation of the placenta in US images. | Copenhagen, Denmark; Astraia database (Munich, Germany) | Government |
| Asadpour, 2023 |  | To propose automated method for processing fetal ultrasonography images to identify placental abruption using ML methods. | California, USA | Foundation |
| Sun, 2022 |  | To introduce PFCnet (placental features classification network), a multi-model model for evaluating and classifying placental features in gestational diabetes mellitus (GDM) and normal late pregnancy. | Shanghai, China | Information not available |
| Wang, 2022 | Segmentation of the placenta only | To propose an improved U-Net framework, RU-Net, for segmenting placental adhesion, implantation, and penetration. | Quanzhou, China | Information not available |

*Small for gestational age (SGA); overlap across multiple categories (*); all objectives were directly adapted from each publication to prevent the original aim of the paper from being misconstrued*

**Other**

11 studies (11%) addressed topics that were not covered by the Horgan categories (Table 3). One study discusses the assessment of amniotic fluid. Two studies focus on Doppler FHR. One study focuses on fetal descent. One study discusses gender blocking. Two studies focus on optimizing standard US. Two studies focus on preterm birth prediction with cervical characteristics. Two studies discuss user accuracy. Of these 11 studies, 6 took place in Asia, 3 took place in Europe, 3 took place in South America, none took place in both North and South America. One study is from a publicly available dataset which did not specify setting. Table S7 further summarizes the publications in this category.

TABLE S7. Other

| **Author, Year** | **Subcategorization** | **Objective** | **Setting** | **Funding** |
| --- | --- | --- | --- | --- |
| *Self, 2022 | Assessment of amniotic fluid | To develop Computer-Assisted Low-Cost Point-of-Care Ultra-Sound (CALOPUS) for nonexpert users to assess fetal viability, detect the presence of multiple pregnancies, evaluate placental location, assess amniotic fluid volume, determine fetal presentation, and perform basic fetal biometry. | UK; India | Government |
| Katebi, 2023 | Doppler FHR | To introduce an end-to-end algorithm applied to a low-cost, hand-held Doppler US device for estimating GA, and by inference, fetal growth restriction (FGR) | Highland Guatemala | Academic  Foundation  Government (4) |
| Valderrama, 2019 |  | To introduce a reproducible and generalizable autocorrelation (AC)-based method for fetal heart rate (FHR) estimation from one-dimensional Doppler US (1D-DUS) signals. | Leipzig, Germany; Oxford, UK; rural highland Guatemala in the vicinity of Tecpán, Chimaltenango | Academic (2)  Government (3)  Private |
| Bai, 2022 | Fetal descent | To propose a new framework for angle of progression (AoP) measurement, including image segmentation, target fitting, and AoP calculation | **Guangzhou and Jinan (JNU-IFM dataset), China** | Academic  Government (3)  Private |
| Lakra, 2019 | Gender blocking | To explore deep learning-based techniques to detect images containing the gender-defining features among the entire set of images in cine-loop, with a conforming accuracy averaging above 80%. | India | Government |
| Huh, 2023 | Optimizing standard US | To enhance the image quality of 3-D US imaging, using a novel unsupervised deep learning approach that aims to overcome inherent resolution limitations observed in 3-D US imaging systems. | Public dataset, geographic setting unavailable. | Academic  Government (2) |
| Wang, 2022 |  | To select US frames for biometry, for which operator skill is assessed by quantifying how well the tasks are performed with neural network-based frame classifiers. | Oxford, UK | Government (2) |
| Andrade, 2023 | Preterm birth prediction with cervical characteristics | To create a new screening for spontaneous preterm birth (sPTB) based on AI. | São Paulo, Brazil | There is no funding associated with the work featured in this article. |
| Ohtaka, 2023 |  | To construct a deep learning model to predict preterm births using transvaginal US images. | Tokyo, Japan | Information not available |
| Clinicaltrials, 2022 | User accuracy | To determine accuracy of US places, completeness of US planes, and estimated fetal weight. | Chengdu, China | Government |
| *Pei, 2023 |  | To develop and validate a fully automated AI system to extract standard planes, assess early gestational weeks, and compare the performance of the developed system to sonographers. | Guangzhou, Zhujiang New Town, and Zengcheng, China | Academic |

*overlap across multiple categories (*); all objectives were directly adapted from each publication to prevent the original aim of the paper from being misconstrued*
